# Changing Malaria Transmission and Implications in China towards National Malaria Elimination Programme between 2010 and 2012

**DOI:** 10.1371/journal.pone.0074228

**Published:** 2013-09-09

**Authors:** Jian-hai Yin, Man-ni Yang, Shui-sen Zhou, Yi Wang, Jun Feng, Zhi-gui Xia

**Affiliations:** 1 National Institute of Parasitic Diseases, Chinese Centre for Disease Control and Prevention (CDC), Beijing, China; 2 Key Laboratory of Parasite and Vector Biology, MOH, Beijing, China; 3 WHO Collaborating Centre for Malaria, Schistosomiasis and Filariasis, Shanghai, China; 4 Guizhou Provincial CDC, Guiyang, China; Menzies School of Health Research, Australia

## Abstract

**Background:**

Towards the implementation of national malaria elimination programme in China since 2010, the epidemiology of malaria has changed dramatically, and the lowest malaria burden was achieved yearly. It is time to analyze the changes of malaria situation based on surveillance data from 2010 to 2012 to reconsider the strategies for malaria elimination.

**Methods and Principal findings:**

Malaria epidemiological data was extracted from the provincial annual reports in China between 2010 and 2012. The trends of the general, autochthonous and imported malaria were analyzed, and epidemic areas were reclassified according to Action Plan of China Malaria Elimination (2010-2020). As a result, there reported 2743 malaria cases with a continued decline in 2012, and around 7% autochthonous malaria cases accounted. Three hundred and fifty-three individual counties from 19 provincial regions had autochthonous malaria between 2010 and 2012, and only one county was reclassified into Type I (local infections detected in 3 consecutive years and the annual incidences ≥ 1/10,000) again. However, the imported malaria cases reported of each year were widespread, and 598 counties in 29 provinces were suffered in 2012.

**Conclusions/Significance:**

Malaria was reduced significantly from 2010 to 2012 in China, and malaria importation became an increasing challenge. It is necessary to adjust or update the interventions for subsequent malaria elimination planning and resource allocation.

## Introduction

Owing to the unprecedented international efforts, malaria mortality rate decreased by more than 25% worldwide between 2000 and 2010, and the estimated incidence rate decreased by 17% in the same period, although malaria remains globally the most important human parasitic disease [1]. In China, malaria seems to have been known for more than 4000 years and was identified as one of the top 5 parasitic diseases that affected seriously the social-economic development after the establishment of the People’s Republic of China in 1949, having the estimated numbers of 1829 (over 70% of the total counties nationwide) malaria endemic counties, more than 30 million cases and about 1% fatality rate in the peak year; afterwards, the reported malaria cases have declined dramatically through years of efforts to less than 15,000 by the end of 2009, the endemic malaria was mainly caused by *Plasmodium falciparum* and *Plasmodium vivax*, and the endemic areas have become proportionally shrinking [2,3].

To effectively protect public health, promote economic development, support achievement of the health-related Millennium Development Goals, as well as the ultimate global goal of malaria eradication, the Chinese government decided to embark upon the national malaria elimination programme (NMEP) in 2010, with a goal of eliminating malaria by 2015 in a majority of the regions with the exception of the border region in Yunnan Province, and to completely eliminate malaria from P.R. China by 2020; and at the same year, the corresponding *Action Plan of China Malaria Elimination* (*2010-2020*) (APCME) was issued officially [4]. In APCME, for sake of better strategy planning and resource allocation, counties in China were stratified into four types (Type I~ IV) based on the malaria epidemic reports of 2006-2008 (Type I: local infections detected in 3 consecutive years and the annual incidences ≥ 1/10,000; Type II: local infections detected in the last 3 years and at least in one year the annual incidence ˂ 1/10,000 and > 0; Type III: no local infections reported in the 3 years; Type IV: non malaria epidemic area.): in Type I counties, the integrated interventions of case management and vector control will be scaled up to reduce the incidence; in Type II counties, disposal of any possible malaria cases and active foci will be the main strategies to interrupt local transmissions; in Type III counties, the capabilities of malaria surveillance and response will be emphasized to prevent malaria reintroduction; and in Type IV counties, the key point is to save lives of imported cases through early diagnosis and appropriate treatment [4].

Through the implementation of APCME with the supports from central and local governments as well as the Global Fund to fight AIDS, Tuberculosis and Malaria, the malaria situation in China changed quickly in terms of malaria cases in recent years and the lowest malaria burden was achieved year by year. The reported malaria cases were reduced by 45.8% in 2010 compared with that in 2009, and continuously 43.0% decline in 2011 compared with that in 2010 [5,6]. The number of nationwide malaria cases in 2011 was no more than 5000. Meanwhile, with the exception of the province of Yunnan, no reported local case of *Plasmodium falciparum* was presented in P.R. China. Hainan Province, one of the most endemic areas with high transmission of *Plasmodium falciparum* historically, hasn’t report autochthonous falciparum malaria cases since 2010 [5,6].

It has been almost 3 years since the APCME was issued, malaria situation in China has changed a lot, and virtually China NMEP also stepped into a new stage. In such a context, in order to provide the requisite decision-making evidence for subsequent NMEP performance, it is the right time to assess progress towards NMEP and update malaria situation based on the surveillance data from 2010 to 2012.

In this paper, it is attempted to reclassify the counties of 4 types using the same criteria in APCME and the updated malaria data between 2010 and 2012, and understand the changes of malaria situation in the past 3 years and challenges in the future. It could provide some potential suggestions to improve the strategies and interventions for eliminating malaria locally and nationally in China.

## Materials and Methods

### Data Collection

Data on malaria cases at the county level between 2010 and 2012 were collected according to the annual submissions from provincial CDCs or institutes of parasitic diseases in China. The data, including province name, county name, population, total number of cases, number of autochthonous cases, and so on, were extracted, and constructed into a new database in the Microsoft excel 2010.

### Classification and Mapping of Malaria Epidemic Areas

Annual incidences of autochthonous malaria at each administrative region between 2010 and 2012 were calculated respectively and used to reflect the extent of local malaria transmission. For each year, all counties in the mainland of China were mapped into four grades according to the autochthonous malaria incidence (≥ 1/1,000, ≥1/10,000 and ˂1/1,000, >0 and ˂1/10,000, 0 respectively). Furthermore, all counties were categorized and mapped according to the principle applied in the APCME aforementioned.

The software ArcGIS version 9.1 was used to describe the spatial distribution at the county level polygon map.

### Statistical Analysis

Using Microsoft Excel 2010 software, the constituent ratio and incidence rate of total, autochthonous and imported malaria were calculated, and their temporal trend and spatial distribution were analyzed. Differences in distribution were evaluated using the chi-square (χ^2^) test by SPSS version 16.0 and *P* < 0.05 was considered statistically significant.

## Results

In general, malaria in China changed dramatically between 2010 and 2012, both the areas and cases suffered from local infection were reduced significantly, and malaria imported became an increasing challenge.

### Counties with Autochthonous Malaria

**Figure 1:**
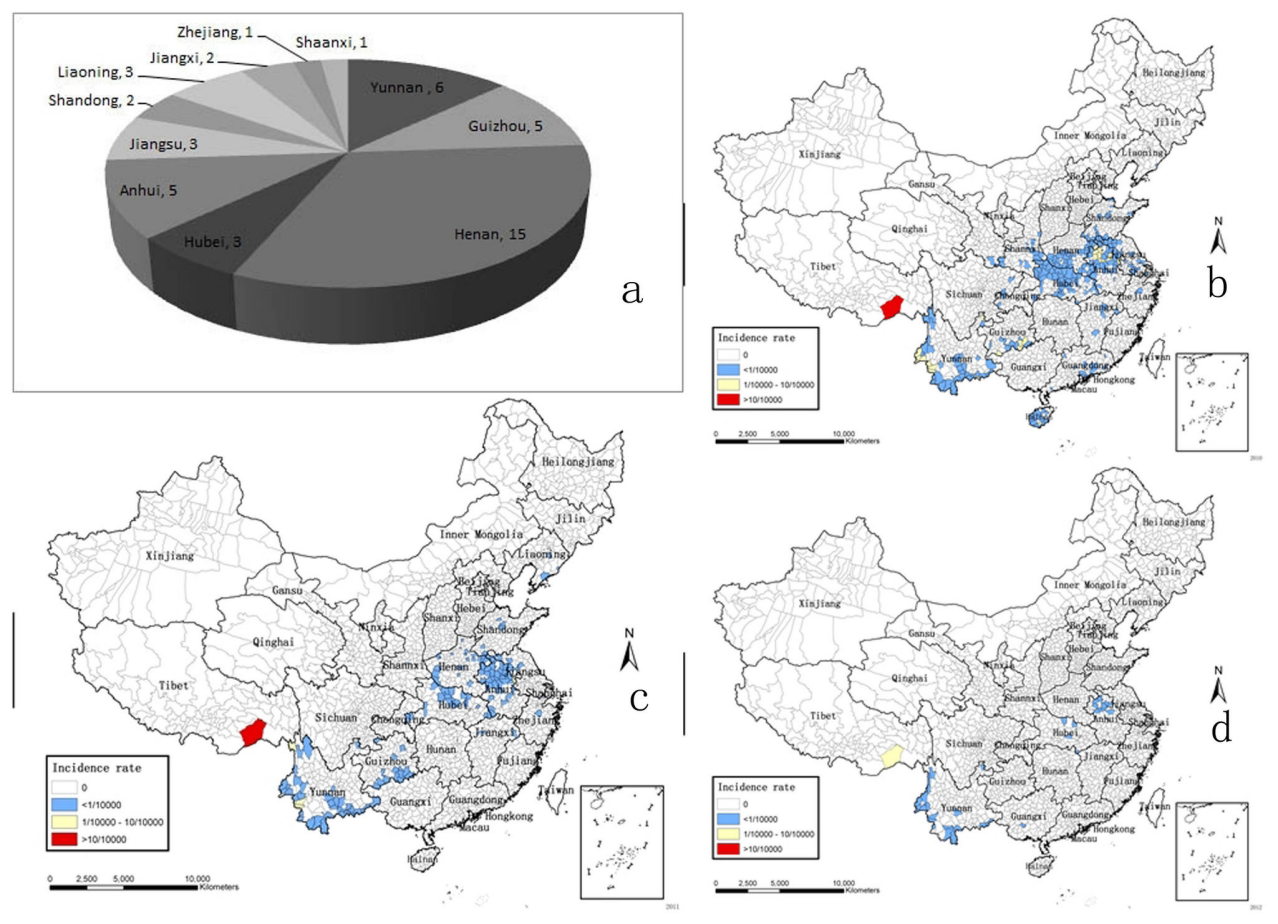
The Shifts of Counties with Different Malaria Incidence Rates. a: distribution of new counties suffered from autochthonous malaria in 2011; b-d: national distribution of counties at four malaria incidence rates in 2010, 2011, 2012 respectively.

In each year, all of the 31 Provinces/Municipalities/Autonomous Regions in the mainland of China reported malaria cases. The areas suffered from autochthonous malaria were significantly shrinking, eighteen provinces and 303 counties in 2010, and twelve provinces and 160 counties in 2011, and 5 provinces and 41 counties in 2012 (*P*=0.003 for provinces, *P*<0.001 for counties; Table 1). However, forty-six new counties from 11 provinces suffered in 2011 compared with 2010 (Figure 1a), four new counties including 3 from Yunnan and 1 from Guangxi in 2012 compared with 2011. In 2012, these 4 new counties were also free from local infections in 2010, and no local infections were reported in the 2011’s new counties. In addition, the intensity of local malaria transmission declined simultaneously. The number of counties with an incidence of ≥ 1/1,000, ≥1/10,000 and ˂1/1,000, >0 and ˂1/10,000 were reduced to 0, 1 and 40 respectively in 2012 (Figure 1b–d).

**Table 1:** Distribution of provinces and counties suffered from autochthonous malaria between 2010 and 2012.

	**Province**				**County**		
	**with local malaria cases**	**without local malaria cases**	**Total**		**with local malaria cases**	**without local malaria cases**	Total*
2010	18	13	31		303	2553	2856
2011	12	19	31		160	2693	2853
2012	5	26	31		41	2811	2852

* the numbers of counties changed due to splitting the combined county and/or combining the divided counties.

**Figure 2:**
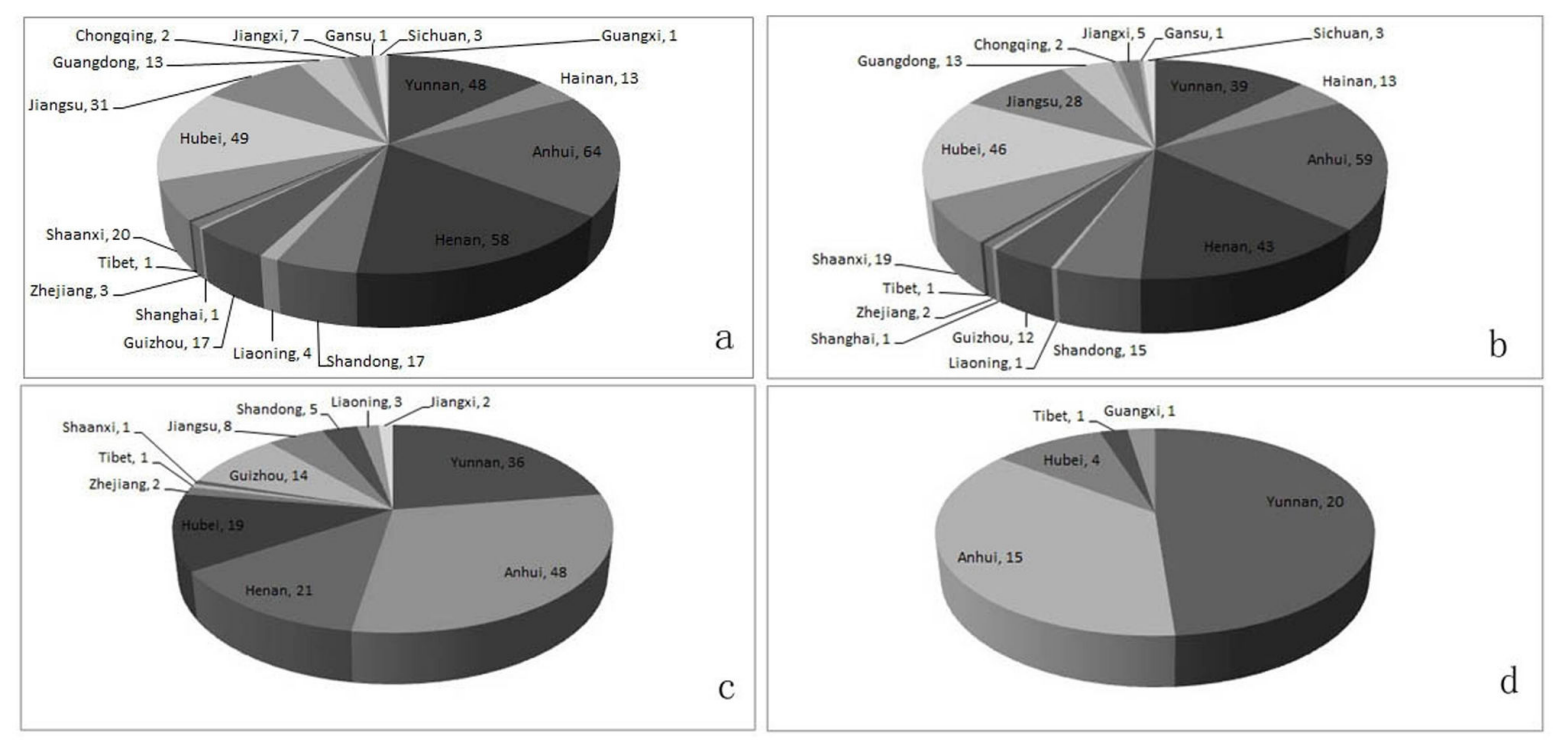
Distribution of Autochthonous Malaria at County Level in the Mainland of China, 2010-2012. a: distribution of total counties suffered from autochthonous malaria,2010-2012; b-d: distribution of counties suffered from autochthonous malaria in 2010, 2011 and 2012, respectively.

Accumulatively, there were 353 individual counties in the 3 years, and the distribution was illustrated in the Figure 2a. Among them, only 15 counties reported were without imported malaria in the 3 consecutive years, eleven from Anhui and 3 from Yunnan and 1 from Tibet autonomous region. The geographical distribution of autochthonous counties each year was shown statistically as Figure 2b–d.

### Category and Mapping of Malaria Epidemic Areas

**Figure 3:**
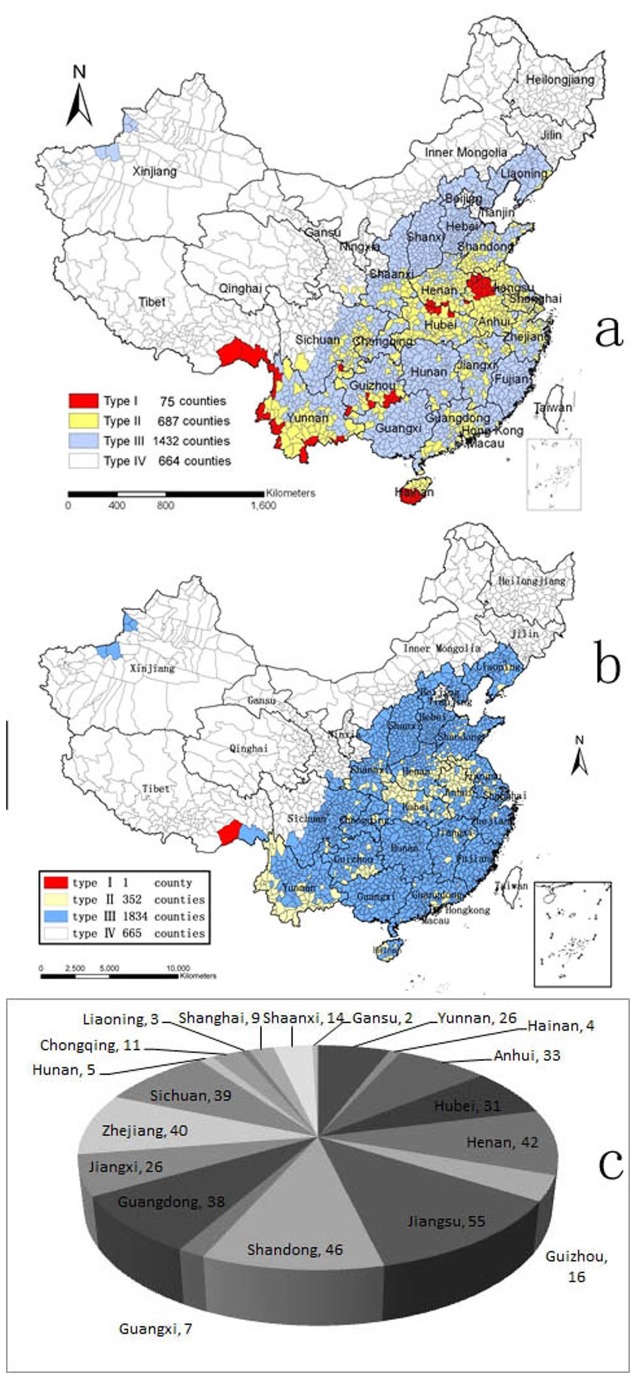
The Shifts of Four Types Malaria Endemic Counties, 2010-2012. a: endemic malaria counties’ distribution in APCME; b: endemic malaria counties’ distribution updated based on data between 2010 and 2012; c: distribution of new Type III counties originated from Type II counties.

All the counties were reclassified using the data between 2010 and 2012 according to the principle in APCME, only one county named Motuo in Tibet remained Type I, it’s annual incidences were 29.83, 16.47 and 8.18 per 10,000 people from 2010 to 2012 successively, especially all the malaria cases reported in this county were autochthonous, and another 352 counties pertained to Type II (*P*<0.001; Table 2 & Figure 3a,b). Obviously, the numbers of counties as Type I and Type II were significantly reduced, and Type III increased. And no Type I counties were located in 6 provinces including Yunnan, Hainan, Anhui, Hubei, Henan and Guizhou again. In detail, the original Type I counties listed in APCME almost changed into Type II, except Luxi in Yunnan and Wuzhishan in Hainan and Chayu in Tibet changed into Type III and Motuo in Tibet remained as Type I. And there were 447 original Type II counties in APCME changed into Type III as detailed in Figure 3c.

**Table 2:** Four types of malaria epidemic counties in two periods.

**County type**		**Type I**	**Type II**	**Type III**	**Type IV** *	**Total** *
2006-2008	75		687	1432	664	2858
2010-2012	1		352	1834	665	2852

* the numbers of counties changed due to splitting the combined county and/or combining the divided counties.

### Imported Malaria

**Figure 4:**
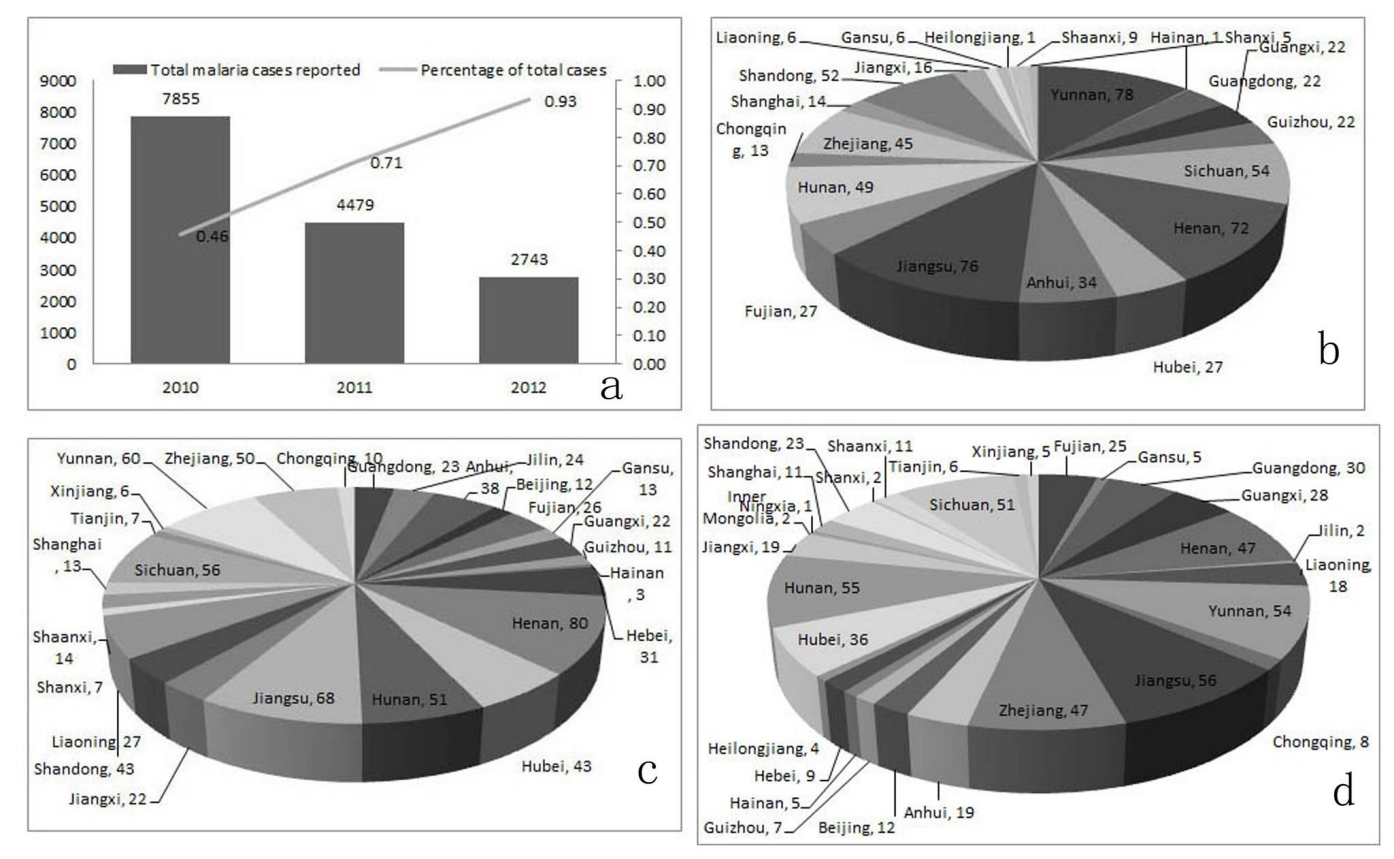
The Changing Distribution of Imported Malaria Cases Reported, 2010-2012. a: imported malaria in the mainland of China, 2010-2012; b-d: national distribution of imported malaria at county level in 2010, 2011 and 2012, respectively.

Compared to the reduced autochthonous malaria, imported malaria has been sustained at a high level, while accounted for a growing share of total malaria cases between 2010 and 2012 in the mainland of China (Figure 4a). Meanwhile, the imported malaria cases were even more widespread. A total of 651 counties in 23 provinces were suffered in 2010, and 760 counties in 26 provinces in 2011, and 598 counties in 29 provinces in 2012 (Figure 4b–d). Most of these cases were suffered from falciparum malaria who came back from African countries or southeastern countries especially Myanmar, and only a small proportion of them were imported from another provinces in China.

## Discussion

Malaria is one of the most important parasitic diseases in China, both falciparum and vivax malaria were historically prevalent with high incidence, but a significant reduction was sustained with the persistent malaria control efforts.

As malaria elimination aims at sustainable interruption of local malaria transmission by mosquitoes despite a continued presence of malaria vector mosquitoes and importation of parasites from abroad through international travel and migration [7], the reduction of autochthonous malaria cases has been targeted by China NMEP in 2010 with the following objectives [4]: 1. to eliminate malaria (no autochthonous cases for at least 3 consecutive years) in all Type III counties by 2015; 2. no autochthonous malaria case in all counties of Type II and Type I (except for some border regions of Yunnan Province) by 2015 and another 3 years to maintain; and 3. autochthonous malaria incidence rate drops to 1/10,000 in Type I counties of Yunnan border regions by 2015, no autochthonous malaria case by 2017, and to nationally eliminate malaria by 2020.

For the NMEP, it is important to periodically review the epidemiology of malaria in the country, to assess progress towards achievements of national targets so that the next steps can be defined to improve programme performance or redefine the strategic direction and focus, including revising the policies and strategic plans [8]. Along with the issue of APCME, malaria situation changed quickly, such a periodic review as WHO recommended is obligatory and beneficial, and thus the present analysis was made.

The data analyzed in this paper were from the annual statistics submitted from local malaria facilities of provincial centers for diseases control and prevention or institutes of parasitic diseases. In view of annual malaria data, it was much more accurate especially due to data was submitted through rechecking step by step at national, provincial and county levels, thus it is suggested that this analysis more reliable to reflect the real malaria situation in China. But there were template modifications for the annual malaria submission in these years; as a result, the detailed species and vectors specific data could not be demonstrated in a unified and consolidated format here.

It is obvious that malaria has been effectively controlled in China since the issue of APCME, the autochthonous cases continuously reduced dramatically between 2010 and 2012, and the range of Type I and II was shrunk accordingly with a patchy and focalized geographical spread (Table 2). It was important that only one county re-ranked as Type I, and no county was categorized as this type in Yunnan and Hainan and central provinces, where were the most severely affected areas historically. Meanwhile, the annual incidence was falling every year. And it reached the target in advance which planed that incidence of Type I counties at border areas of Yunnan should be reduced to less than 1/10,000 by 2015 [4]. As well as it promoted the malaria elimination programme in other type counties steadily.

The guiding ideology of categorized and localized malaria control has been proved as the successful and valuable experience in China. Accordingly in response to such a rapid changing malaria situation of current China, a direct suggestion is that each area should make the corresponding adjustments of their strategies and tasks based on their newly stratified position. The original Type I and Type II counties don’t need to take the high coverage of interventions that will be not cost-effective, but more targeted, and refined interventions as well as sustainable coverage of good-quality laboratory and clinical services, reporting and surveillance must be reinforced.

Although the malaria in China has been effectively controlled particularly after the implementation of the strategy consisting of one day to report the case, three days to investigate it, and seven days to finish a foci response, the experiences are still under revealing the exact effective interventions-the social-economic development, climate changes or other ecological factors. But some successful experiences from the pioneer provinces can be introduced to the current 41 autochthonous malaria counties as well as Type II counties for malaria elimination.

Moreover, it was found that there were some unstable counties with autochthonous malaria (Figure 1a), the reason was uncovered to date completely [9]. It reminds us of paying some attention to these areas in order to prevent the potential re-emergency or outbreaks just like vivax malaria happened in central China [10,11]. Vivax malaria has been predominant in China, and temperate climate *P. vivax* has a long incubation time and could be a re-emerging threat [12]. The tools for its distinguishing and treatment seem very important [13]. At least, those counties with re-emerging autochthonous malaria have to start all over again to eliminate it and maintain another 3 years.

Besides, malaria importation posed a constant threat to the mainland of China in these 3 years, the proportion increased year by year (Figure 4), composing of not only dominant falciparum malaria from Africa countries, dominant vivax malaria from Asia countries, but also some malariae malaria and ovale malaria cases [6]. It could be attributed to humans or mosquitoes carrying malaria from endemic areas across international boundaries or within countries. As the malaria transmission setting cannot be changed fundamentally as malaria cases decreased, it caused an important question that whether mosquitoes in China could be infected subsequently with *Plasmodium* spp. imported and lead to malaria transmission. And with the continuous globalization, more and more workers in China nationwide are exported to Africa, as well as the frequent population movements in the border areas with Myanmar, it is necessary to intensify the surveillance and management of population back from or from malaria endemic countries and areas with timely detection, diagnosis and appropriate treatment in time.

And refer to malaria vectors, four main kinds of anopheles such as *Anopheles* *sinensis* , *Anopheles* *anthropophagus* , *Anopheles minimus* s.l., *Anopheles dirus* s.l. with individual habitat were recorded in different areas of China. However, continuous and systematic data were rarely updated about their epidemiology [14,15]. And little data about insecticide resistance [16–18] and mosquitoes behavior changes [19] were reported. All the above are very useful for malaria control, and even necessary for malaria elimination and its certification [20]. Therefore, relative studies about the biology, ecology and transmission risks of malaria vectors should be recognized. Such as, the role of the increased urbanization in China should be expressed, whether it leaded to vector redistribution or malaria declines [21]; whether the insecticide use has changed the mosquito behavior that biting times and phenology altered [22]; whether there existed the potential risk factors for malaria re-emerging such as Greece [23], and so on.

There are other critical issues: in low transmission settings, how to identify asymptomatic infections, how to detect accurately individuals with micro-parasitaemic infections, and how to achieve a sufficient sample size to have an adequately powerful study [24], etc. What’s more in China, there was a gap between the requirements of laboratory testing rate of malaria cases and the rate of laboratory confirmation as some clinically diagnosed or unconfirmed cases still existed [25]. It is necessary to improve the development of malaria diagnosis reference laboratories for malaria confirmation, and clinicians even from medical institutes of village level should receive much more trainings on malaria diagnosis and treatment, and sensitive and specific and inexpensive techniques should be used [6,25].

The changing epidemiology and its complexity of malaria elimination has become more apparent, and China is facing the same challenges as dozen of malaria-eliminating countries in the world, approaches to elimination need to be aligned with these changes, the novel strategies and methods that address the changing epidemiology need to be developed, validated, and adopted [26–28]. Based on the present analysis, speciﬁcally in China, the possible acting points could be suggested as: 1. programme reorientation with localized, sustainable and cost-effective interventions; 2. instability management in original endemic counties particularly having vivax malaria; 3. systematical vector surveillance in behavioral changes, plasmodium infectivity, vectorial capacity and insecticide resistance; 4. improve the capacity to stop imported malaria by integrated interventions that covers all aspects from case detection, vector control and international cooperation and so on; 5. deepen malaria “T3: Test. Treat. Track” approach, and perfect the sensitive surveillance and effective response system [29].

However, the goal of elimination only can be reached if we could timely change the concepts, seize those opportunities and deal with challenges, as well as accelerate the technique innovations and breakthrough by scientific researches [30,31]. It is more critical.
